# Experiencing and enduring patient distress: the distress of palliative care patients and its emotional impact on physicians in training

**DOI:** 10.1186/s12909-024-05668-9

**Published:** 2024-06-26

**Authors:** Andréa Tarot, Maxence Pithon, Ashley Ridley, Virginie Guastella, Morgane Plancon, Régis Aubry, Helène Vaillant Roussel, Axelle Maneval

**Affiliations:** 1 Palliative Care Unit; CHU Louise Michel, Palliative Care Center of the Clermont-Ferrand University Hospital, 61 Route de Chateaugay, Cébazat, 63118 France; 2 ACCePPt Research Laboratory at the University Clermont Auvergne, 28 Place Henri Dunant, Clermont-Ferrand, 63000 France; 3 Department of General Practice in Clermont-Auvergne University, 28 Place Henri Dunant, Clermont-Ferrand, 63000 France; 4 ACTé Research Laboratory at the University Clermont Auvergne, 36 Avenue Jean-Jaurès, Chamalieres, 63407 France; 5 Neurodol Research Laboratory at the University Clermont Auvergne, 28 Place Henri Dunant, Clermont-Ferrand, 63000 France; 6 Necker Children’s Hospital, 149 Rue Sèvres, Paris, 75015 France; 7 Palliative Care Mobile Unit – General Hospital, Avenue Désandrouin, Valenciennes, 50479 France; 8 Laboratory of Neurosciences/EA 481 UBFC, University Bourgogne Franche-Comté, 2 place du Maréchal Leclerc, Besançon, 25030 France; 9 Bourgogne Franche Comté Inter-Regional Ethical Reflection, Besançon, France; 10 French National Consultative Ethics Committee, Paris, France

**Keywords:** Medical psychology, Palliative care, Psychological distress

## Abstract

**Background:**

The extreme vulnerability experienced by patients in palliative care may result in significant distress. These patients require appropriate care while not pathologizing their natural distress. Given the challenges of caring for people experiencing significant distress, it is important to understand what professionals in training may feel when caring for patients in palliative care. Therefore, the aim of this study was to explore how professionals in training feel when confronted with the distress of patients undergoing palliative care.

**Methods:**

A qualitative study employing interpretative phenomenological analysis was conducted. In 2022, 11 physicians in training were interviewed about their experiences with distressed patients due to palliative care. The interviews were conducted via video conference. The students participated in the national palliative care cross-training and were in their final year of residency training.

**Results:**

The interviews revealed the following five themes: feelings of powerlessness, duty to act, difficulty in building a relationship, feeling insecure about oneself, and creating a space for listening and relating. All participants felt powerless in front of their patient’s distress. Numerous defense mechanisms were identified that made the relationship with the patient difficult. Four participants described being able to create a space for listening and relating to their patients.

**Conclusions:**

A minority of students could establish a quality relationship with their distressed patients. Two concepts, interprofessional education and the patient-centered approach, were identified and could be developed in training.

## Background

In 1997, the National Comprehensive Cancer Network (NCCN) defined distress as a “multifactorial, unpleasant experience of a psychological, social, spiritual, and/or physical nature that may interfere with the ability to cope effectively with cancer, its physical symptoms, and its treatment. Distress ranges along a continuum from the common normal of vulnerability, sadness, and anxiety to problems that can become disabling, such as depression, panic, social isolation, and existential and spiritual crisis” [1]*.* This definition helps us avoid reflexively pathologizing the distress of patients receiving palliative care and instead allows us to consider their experience as a response to a situation of extreme vulnerability [2, 3].

Even if this experience is not pathological, it requires specific and appropriate management. In this regard, the NCCN developed recommendations for distress management in 2019 [4]. According to these recommendations, the management strategy for such patients is complex and multidisciplinary, with no specific drug treatments. These recommendations imply that the physician’s role is to diagnose and direct the management of patients in distress differently from the management of patients with a psychiatric pathology. However, no studies have addressed whether physicians are trained to do this. Some studies have shown that the emotional experiences of physicians can influence their decision-making [5]; therefore, it is important to understand what professionals in training might feel when confronted with patients’ distress in palliative care.

If specific recurring emotions are identified among professionals in this setting, educating future caregivers about them could help them integrate their experiences to inform future patient management.

## Methods

This was a qualitative study embedded in a constructivist paradigm. We adopted phenomenological interpretive analysis, which allows us to examine how a life experience was perceived and interpreted by the individual who lived it. It assumes that researchers will use their own subjectivity as an investigative tool. The reality being approached is then constructed by the individuals, rather than existing in a fixed form, which aligns with the constructivist paradigm [6, 7]. This study was conducted as part of the thesis project for a Master of Science (MS) program in palliative medicine research. The pedagogical committee, as well as the students, contributed to the study design.

### Recruitment

Our study focused on students in the final year of residency training and enrolled in the national palliative care cross-training for 2021–2022. An invitation to participate in the study was sent to the entire class via email. A second follow-up email was sent 1 month later. The participants were interviewed between March and June 2022. They were offered a semi-directed interview by videoconference, recorded with their consent. We stopped recruiting when we determined that we had reached data saturation, as assumed and discussed among the researchers ^6.^

### Data collection and analysis

The interview guide is consistent with the interpretive phenomenological analysis and is non-rigid to facilitate exchange [8]. It was tested with one participant before beginning the interviews. No modifications were made during the interviews, but the follow-up questions used varied according to the participants. During the semi-structured videoconference interviews, the participants were asked to describe a significant situation that they had experienced with a palliative care patient in distress. Thereafter, they were asked about their feelings toward the patient.

Out of a class of 51 students, 12 participants were included in the study. One participant discontinued the national palliative care cross-training; their interview was non-contributory because they did not have a situation to relate to. Eleven interviews were analyzed; the participants comprised two men and nine women. Their specialties included geriatrics (*n* = 2), oncology (*n* = 1), public health (*n* = 1), and general practice (*n* = 7). The interviews lasted from 19 min (shortest) to 34.20 min (longest).

All interviews were conducted and transcribed verbatim by author AT –a physician in the palliative care unit and a student in the MS program. Data coding was performed in several steps and according to an interpretive phenomenological analysis of the interviews. First, descriptive line-by-line coding was performed using coding software (Nvivo, RITME, French) to identify the initial codes. A triangulation of the data from line-by-line coding to the initial codes was performed separately for the first two interviews with physicians in the palliative care unit (MP and AM). Then, the initial codes were developed into preliminary themes with the study group. A final list of the themes and categories was developed by RA and AM. Preunderstanding could be worked on with the multidisciplinary Master's study group (physician, psychologist, nurse) to deconstruct the researcher's expectations and remain open to unexpected results [9].

The study was declared to the data protection officer at the Clermont-Ferrand University Hospital (French, Clermont-Ferrand). The study was conducted according to the MR004 reference methodology. The Clermont-Ferrand University Hospital has signed a commitment to comply with this “reference methodology” dated March 29, 2022. This study was registered in the establishment’s treatment register under the number M220402.

## Results

**Fig. 1:**
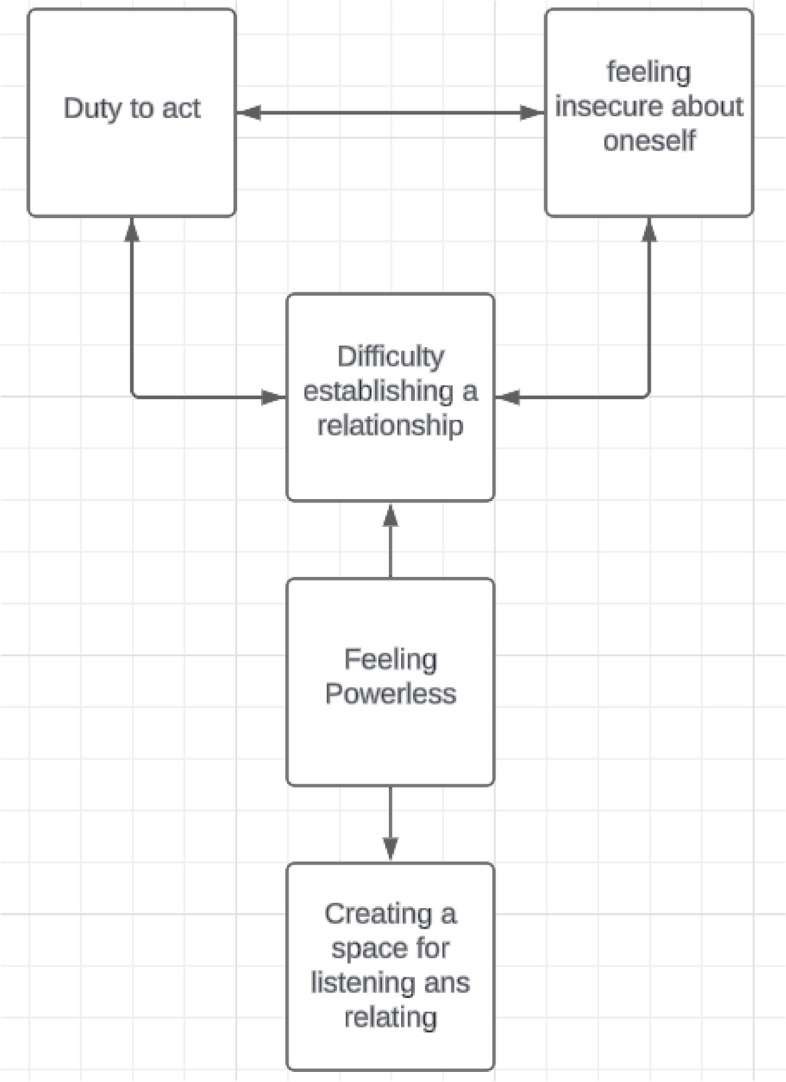
Five emerged themes

The following five themes emerged: feeling powerless, duty to act, difficulty in establishing a relationship, feeling insecure about oneself, and creating a space for listening and relating (Fig. 1).

### Feeling powerless

The participants described feeling powerless at the beginning of their interview. In most cases, the powerlessness was expressed with the pronoun “we.” They describe a feeling of powerlessness in the face of the specific situation of patient distress. This does not mean that they are generally powerless; powerlessness is not a personality trait but a specific feeling in this situation.

It was quite frustrating for me not to be able to help him, to feel a bit powerless. Because in the end, we had reached the end of our therapeutic possibilities. (Student 10)

A powerlessness... it was the powerlessness that made us uneasy, I think. There is a psychologist who told me the other day that there are two things that break down people’s psyches: death and powerlessness, the fact of being incapable. It’s a bit like what happens when you see a traumatic scene, you see a terrible thing and you can’t do anything. And here, I think, we are a bit in it, we are in a kind of collective trauma, feeling powerless before this patient. (Student 12)

And we...for us, the psychological suffering was at first sight, we were not able to work with him in this sense, and therefore this created for us a great frustration... a terrible experience of powerlessness. (Student 7)

When the feeling of powerlessness was evoked, it was often a question of helplessness, of loneliness, and of having to do things alone:

The great helplessness and... I would also say loneliness too...being alone with this patient. And I felt very, very helpless and very concerned about his distress. (Student 11)

With the notion of solitude, the participants used the article "we" to refer to a larger entity in order to recreate reference points. The sense of powerlessness was closely related to the next theme: the duty to act. The sense of powerlessness called for action. Most of the time, “feeling powerless” was not expressed verbally at the moment but was articulated later in the interview, using the same terms as those used to describe the feeling of powerlessness.

So what drove us was again the frustration, the fact that we wanted to succeed, we wanted to bring him well-being, whatever the form of well-being, it was so difficult for us, that we were ready for many things. (Student 12)

All that to say, to summarize, a powerlessness..., in fact, it is the powerlessness that made us so bad, I think, and that’s it. (Student 12, later in the interview).

### Duty to act

The duty to act was expressed by the participants. They were able to verbalize the need to act in response to patient distress. Action was necessary, despite the description of it as ineffective. However, it was not carried out with the patient's outcome in mind.

The mass continued to grow and so he had intestines coming out of the stoma opening into the pouch. I couldn’t tell if it was anxiety or physical pain, but sometimes seeing his intestines in the pouch could cause a crying fit, screaming... distress, and at that point I would feel obligated to do something, so I would bolus and the boluses wouldn’t work... (Student 11)

The participants could perceive an “obligation to do” with several notions of “good” and “bad” in the discourse to justify this transition to action. This “obligation to do” was very much linked to a moral obligation. This theme was closely linked to the notion of value, with an overexpression of the superego in the participants.

Because...on the one hand I thought it was good because I felt like I was doing the right medicine, and on the other hand when I heard the request, I was a little...I thought I shouldn’t have... (Student 4)

Typically, the patient in pain in my mind is not good. And so that means that my job is to make sure that he doesn’t stay in pain. (Student 6)

I think that’s a little bit my “physician” side, but it’s true that I’ve noticed that there’s a lot of desire to respond... well to respond to that distress by trying to... for example, I find it easier to deal with a physical symptom like pain or even anxiety or congestion... because... bang, we can pull things out of the bag. (Student 8)

### Difficulty establishing a relationship

The participants described interactions with patients as unsatisfactory or at least perceived that the interactions were not optimal.

She was a woman that I had difficulty establishing a relationship with because she was well known in the department, I’m just a trainee, just passing through, and she had trouble accepting that the team was helping her, she was so nice but we felt that there was still... she was quite closed off. (Student 10)

I felt that I was not fully in tune with my patient and that I was not listening enough. (Student 7)

The participants felt less capable or unable to listen attentively to distressed patients. They felt the exchange remained superficial, sometimes out of tune with the patient’s situation.

I did not allow myself to break through this veneer. I knew that there were members of the team, like the psychologist or certain members of the team, but there were not many of them, contrary to the usual situation where the team, well... of course the first place is for the family, but there is always a bit of closeness, but there I did not, I really stayed on the surface. In the symptoms, “Are you relieved or not?” But I had a hard time going...because there was clearly pain uh...a psychological dimension to the pain as well uh...physical integration that was...so I stayed pretty much on the surface. (Student 10)

When the professional could not describe their difficulties in the relationship, in most cases, they were left with a great deal of incomprehension with respect to the patient. The participants described communication with uncooperative patients as a power struggle between the professionals and the distressed patient.

Despite the restraint, there is sometimes a bit of anger because it was like confronting a wall. (Student 1)

There may have been a little bit of anger, we have to be a little bit honest too, but I admit that at the very end it was despair, and then, the total lack of satisfaction throughout the care. There was nothing satisfying, nothing at all! We tried everything, there are even things we did for her that we never did for other patients, we went out of our way to get her organic fruit, to squeeze her..., we never did..., she had a treatment..., well in my opinion, and also in the opinion of other people, she still had a preferential treatment..., we gave everything. And in fact, it took us long weeks, even several months, to understand that in fact, no matter what we gave her, no matter how much effort we made, never a thank you, never, never a satisfaction, nothing! (Student 12)

The participants describe numerous defense mechanisms, in particular repression and projection. They were no longer "the bad doctor"; rather, the patient became the "bad patient."

### Feeling insecure about oneself

The participants described feeling a sense of danger towards the end of the interview. This danger was not explicitly identified but was often described as a feeling of insecurity about oneself. The description of the danger itself by the participants was highly fantasized and never precisely described. Paradoxically, death was absent from the participants' discourse.

I had a kind of dissociation in front of me and myself during the interview I found myself a little bit torn between “listen to him, be the most...you are asked to be the most...you have to remain reassuring for this patient” and at the same time telling myself “don’t be too much of a pushover either...”, I felt torn, I really felt it at one point during the week when it went to a crescendo. I didn’t feel very stable in interviews. I didn’t feel safe myself in my relationship, in my therapeutic relationship. (Student 7)

I have the impression sometimes that it also allows me to protect myself in a certain way because in fact when you start to... it’s a degree of suffering that is so great that if you get too close to it, you suffer too. I have the impression that, in general, these are situations that often take a lot of energy, that require a lot of...well, we often discuss it again afterward among ourselves or in the staff because we can’t handle it alone. (Student 8)

I didn’t know to what extent I could be affected, and so I wasn’t able to keep the necessary distance... the small distance that is necessary. I think I put up the barrier and I kept it up until the end because... yes, I was afraid of myself at that point, of my reaction. (Student 11)

This feeling was intensely expressed using strong words: “I had to, completely, too much…”. The danger was also described as intruding at the psychic level, making it difficult to maintain boundaries between the private and professional spheres.

I brought it back a little bit in the private sphere, my companion who is not a physician at all, not at all... Normally I compartmentalize the work quite a bit, personally, but there I admit that it is something that I was not able to leave at work. (Student 12)

Let’s say that it was a case that was of concern beyond the patient’s room. It was something that occupied the mental space, a little bit beyond what it was confined to. (Student 3)

### Creating a space for listening and relating

Four out of the 11 participants described a shift in their interactions with the patient, marked by a transition to active listening that facilitated new insights during the interview. These moments of interaction fostered a unique relationship between the patient and the professional, providing space for both parties to communicate, thereby advancing patient understanding and management through a collaborative process.

So, I think both because we allowed him, well the way we allowed him to build that relationship, because we actually asked him questions that were also personal. We also tried to understand a lot of things. It’s also our attitude to have been available, to have spent time, and in most cases it’s a little bit the framework that is given in the sense that we have this time that we don’t usually have in the specialized services. And so, I think it’s a little bit on both sides, he invested a lot of time because that’s why it left an impression on me, but it’s us who gave him that time and that way of investing it, so he just occupied the space that we had given him. (Student 3)

Little by little, this is a lady that I saw several times, in different contexts, sometimes in a consultation, in the emergency room during one of the shifts, in a certain context, and then over time went by she became a lady, we got attached to her, there are always patients that we get attached to more or less, and she was a lady that we had more or less... yes, she was a lady that was pleasant. We made a commitment to follow her from the beginning, we said “well listen to us as soon as you come, we’ll be there to see you if you feel like it” and then she said yes to us, so somewhere along the line we weren’t at a dead end because I think this lady was being helped. (Student 9)

These participants could describe a very special relationship with distressed patients, highlighting a relational space that emerged during the exchange, enabling the patient to invest in the relationship. The interviews revealed few misunderstandings, and the participants perceived the exchanges as high quality. Detailed information about the patients, including their history and the content of the interviews, was provided by all of these four participants, each expressing satisfaction with the care provided to these patients.

That’s also what’s interesting because I think that’s what... that’s the space where most of the stuff happens. And in some ways, it’s almost the most interesting care because there’s a lot of personal stuff going on, a lot of what’s being delivered and their experiences. Ultimately, there is something that comes out of these treatments that is extremely rich. And it doesn’t happen very often, and when it does, we’re happy that it can happen, it means that we’ve done what was possible. There is also this idea that it is almost a success to have been there for these patients in distress. (Student 3)

We can see here that the participants were aware of what they had been able to put in place to ensure that the relationship was of high quality, and this was rewarding for them.

## Discussion

The participants in our study revealed that they experience difficulties in their relationships with distressed patients in the vast majority of cases. They all felt a sense of powerlessness, which led them to use some defense mechanisms to protect themselves from this feeling [10]. Their initial training did not prepare them to understand these feelings of powerlessness.

The North American Nursing Diagnosis Association defines helplessness as “Pa feeling that one’s actions will have no effect, a feeling of being helpless in the face of a current situation or immediate event” [11]. This definition highlights the connection between feelings of powerlessness and action. It also qualifies that this feeling is an impression, as in Miller’s definition of powerlessness [12]. Its description refers to the feeling experienced by a person in relation to the circumstances of the moment or generated by the given situation itself. Therefore, powerleness does not imply a stable personality trait. In this sense, powerlessness is an existential construct conditioned by a specific situation with which the person is confronted. Professionals who can create a space for listening and relating to the patient understand this nuance and can perceive the effectiveness of their care. By contrast, those who cannot understand this nuance or perceive the effectiveness of their care may become victims of the feeling of powerlessness. They describe a strong sense of helplessness with a loss of direction and a feeling of loneliness. In these situations, the pronoun “we” suggests unity. In addition, to alleviate this experience, the participants felt the need to act, even if it was considered useless. This is confirmed by Simon’s work in the 1950s, highlighting the limits of the rational decision-making approach [13].

Only four participants could create a space for listening and relating that was comparable to the patient-centered approach described by Roger [14, 15]. These participants felt less powerlessness than the other participants did and created a special space with the patient. This space seems to be beneficial for both the patient and the professional. Compared with the other professionals, these professionals appear to be in less pain, satisfied with the care given, and feel valued. This space allows co-construction; the professional strives to “understand” the patient and who they are entirely. Finally, focusing on understanding the patient and establishing a caregiver–patient relationship helps professionals shift from their own experience to that of the patient. This space for dialogue becomes a useful therapeutic tool for the healthcare professional.

Creating a space for listening and relating to the patient seems necessary to successfully support distressed patients in palliative care. We propose two concepts to help professionals create this space: interprofessional education (IPE) and the patient-centered approach.

### Interprofessional education

The evolution of medicine today leads to increasingly complex situations; this partly explains the need to involve several professions in the management of patients [16]. The IPE is a very interesting concept to help professionals work together [17], especially in the palliative context [18]. The World Health Organization defines IPE as “when two or more professions learn about, from and with each other to enable effective collaboration and improve health outcomes” [19].

In the specific situation of a distressed patient in palliative care, interdisciplinarity would offer a physician in training an analysis of their subjectivity through a joint interview with the psychologist of their team to elaborate on the feeling of powerlessness as a mirror of the patient’s distress. Psychologists in palliative care have already described this role in France since 2016 [20, 21]. Interdisciplinarity would also help address the second concept, the patient-centered approach. The patient needs a physician working as part of a team to be understood as a whole. Each brings a unique viewpoint, allowing for a global patient understanding.

### Patient-centered approach

The theme of “creating a space for listening and relating” echoes Roger’s concept of the patient-centered approach [15]. Interestingly, this theme can be related to a known concept that can be taught and explained. Several studies show the importance of a patient-centered approach in general practice [22] and palliative care situations [23]. A 2017 study showed good results regarding the effect of a patient-centered communication intervention on oncologist–patient communication and quality of life [24]. In this study, a brief combined intervention for physicians and patients with advanced cancer promoted patient-centered communication in the short term, with clinically significant increases in patient engagement in emotional response and discussions about prognosis and treatment choices. This approach would improve the quality of professional relationships with distressed patients in palliative care.

It would be interesting to continue this work by studying the effect of introducing these two concepts to students dealing with patient distress.

### Strengths and limitations

One of the limitations of this study lies in the inclusion of the principal investigator in the analysis; the investigator is a palliative care physician who is in daily contact with distressed patients in palliative care. Their involvement in daily patient care might have introduced bias in data analysis.

Another limitation is the short duration of the training program for the interviewees (i.e., 1 year). Furthermore, the study population was predominantly female.

Regarding the study’s strengths, the study population is geographically distributed throughout France; therefore, it is representative of all palliative care services in the nation. The interviews provided substantial data, allowing for a rich analysis of the participant’s experiences and related feelings. Finally, the study’s subject was of interest to the participants: all could relate with interest to a situation they had experienced during their journey. Thus, students in palliative care medicine may find the subject of this study interesting.

## Conclusions

Even though this is a small-scale study, its findings demonstrate that residents may establish a quality relationship with their distressed patients, although some residents were capable of this. Therefore, factors that can help professionals establish quality relationships with their patients must be identified. Two potential leads may be the existing concepts of IPE and the patient-centered approach, which could help students confront patient distress. Furthermore, with this study, we hope to raise awareness of the participants’ observed defense mechanisms to enable students to move from defense to coping mechanisms, in order to support their own well-being and improve the quality of their patient care.

## Data Availability

The datasets used and/or analyzed during the current study are available from the corresponding author on reasonable request.

## Abbreviations

IPE: Interprofessional education
NCCN: National Comprehensive Cancer Network
MS: Master of Science

## References

[1] National Comprehensive Cancer Network (2003). Distress management. Clinical practice guidelines. J Natl Compr Canc Netw.

[2] Lloyd-Williams M, Reeve J, Kissane D (2008). Distress in palliative care patients: developing patient-centred approaches to clinical management. Eur J Cancer.

[3] Van Lander A, Tarot A, Savanovitch C, Pereira B, Vennat B, Guastella V (2019). Assessing the validity of the clinician-rated distress thermometer in palliative care. BMC Palliat Care.

[4] Riba MB, Donovan KA, Andersen B, Braun I, Breitbart WS, Brewer BW (2019). Distress management, version 3.2019, NCCN clinical practice guidelines in oncology. J Natl Compr Canc Netw..

[5] Ondet M, Poulet C (2020). Prise de décision d’une LAT des médecins exerçant en soins palliatifs: éthique et vécu émotionnel. Médecine Palliat.

[6] Smith JA. Qualitative psychology: a practical guide to research methods. London: Sage Publications; 2015. p.29.

[7] Smith JA (2004). Reflecting on the development of interpretative phenomenological analysis and its contribution to qualitative research in psychology. Qual Res Psychol.

[8] Nizza E, Farr J, Smith JA (2021). Achieving excellence in interpretative phenomenological analysis (IPA): four markers of high quality. Qual Res Psychol.

[9] Palmér L, Nyström M, Ekeberg M, Lindberg E, Karlsson K (2022). Pre-understanding—a threat to validity in qualitative caring science research?. Int J Hum Caring.

[10] Freud A. The ego and the mechanisms of defence. 1st Edition. New York: Routledge; 1966.

[11] NANDA International. Nursing diagnoses 2012-14: definitions and classification. John Wiley & Sons; 2011.

[12] Miller JF. Coping with chronic illness: overcoming powerlessness. 3rd ed. Philadelphia: F.A. Davis Company; 1983.

[13] Simon HA (1957). Models of man: social and rational; mathematical essays on rational human behavior in society setting.

[14] Rogers CR. On becoming a person: a therapist’s view of psychotherapy. Boston: Houghton Mifflin Harcourt; 1995.

[15] Rogers CR (1986). Carl Rogers on the development of the person-centered approach. Person Cent Rev.

[16] Lumague M, Morgan A, Mak D, Hanna M, Kwong J, Cameron C (2006). Interprofessional education: the student perspective. J Interprof Care.

[17] Hammick M, Freeth D, Koppel I, Reeves S, Barr H (2007). A best evidence systematic review of interprofessional education: BEME Guide no. 9. Med Teach.

[18] Kirkpatrick AJ, Donesky D, Kitko LA (2023). A systematic review of interprofessional palliative care education programs. J Pain Symptom Manage.

[19] Gilbert JH, Yan J, Hoffman SJ (2010). A WHO report: framework for action on interprofessional education and collaborative practice. J Allied Health.

[20] College of psychologists of the French society for palliative care support. Referential of the practices of psychologists in palliative care. Published online June 2016.

[21] Maneval-Van LA (2023). Référentiel international francophone des pratiques cliniques des psychologues en soins palliatifs. Revue Internationale de Soins Palliatifs.

[22] Hashim MJ (2017). Patient-centered communication: basic skills. Am Fam Physician.

[23] Ngo-Metzger Q, August KJ, Srinivasan M, Liao S, Meyskens FL (2008). End-of-life care: guidelines for patient-centered communication. Am Fam Physician.

[24] Epstein RM, Duberstein PR, Fenton JJ, Fiscella K, Hoerger M, Tancredi DJ (2017). Effect of a patient-centered communication intervention on oncologist-patient communication, quality of life, and health care utilization in advanced cancer: the VOICE randomized clinical trial. JAMA Oncol.

